# Characterising apathy and anhedonia in dementia using digital behavioural biomarkers – Paving the way for therapeutic monitoring in the home setting

**DOI:** 10.1002/alz70856_096683

**Published:** 2025-12-24

**Authors:** Julie Behenska, Claire Elise Berner, Elena Karpinski, Candice Gautier, Monica Toba, Idil Sezer, Valérie Godefroy, Raffaella Migliaccio, Richard Levy, Karim N’Diaye, Muireann Irish, Bénédicte Batrancourt

**Affiliations:** ^1^ Sorbonne Université, Institut du Cerveau ‐ Paris Brain Institute – ICM, Inserm, CNRS AP‐HP, Hôpital de la Pitié Salpêtrière, Paris, Ile de France, France; ^2^ Brain and Mind Centre, School of Psychology, The University of Sydney, Sydney, NSW, Australia; ^3^ Laboratory of Functional Neurosciences (UR UPJV 4559), University Hospital of Amiens and University of Picardie Jules Verne, Amiens, Hauts‐de‐France, France; ^4^ Université Claude Bernard Lyon 1, CNRS, INSERM, Centre de Recherche en Neurosciences de Lyon CRNL U1028 UMR5292, Bron, Rhône‐Alpes, France

## Abstract

**Background:**

Despite growing interest in Behavioural and Psychological Symptoms in Dementia (BPSD) there remains a lack of reliable methods to accurately monitor these disturbances in home settings. This study focuses on two prominent BPSDs ‐ apathy (i.e., a reduction in goal‐directed behaviour) and anhedonia (i.e., a decreased ability to experience and to pursue pleasure). Although distinct, these symptoms often co‐occur in patients with Alzheimer's disease (AD) and behavioural variant of frontotemporal dementia (bvFTD), significantly undermining patients' autonomy and quality of life. Validated and reliable markers of these symptoms are needed to ensure reliable disease monitoring and to inform the development of targeted interventions.

**Methods:**

The ECOCAPTURE@HOME study (Clinicaltrials.gov: NCT04865172) aims to remotely measure behavioural markers of apathy such as daytime activity, quality of sleep, emotional arousal, in everyday life. Here we extend this framework to explore the utility of digital markers in capturing apathy and anhedonia in the home setting in dementia. Spanning two sites, the Paris Brain Institute and the University of Sydney, we aim to recruit 20 AD patient‐caregiver dyads, 20 bvFTD patient‐caregiver dyads and 20 healthy control dyads. Empatica EmbracePlus (Empatica Inc., Boston, MA, USA) wristbands equipped with multiple integrated physiological sensors (e.g., accelerometer, electrodermal activity sensor), alongside targeted behavioural questionnaires and ecological momentary assessments will be implemented. Machine learning methods will be used to explore profiles of apathy and anhedonia at the group and individual patient level.

**Results:**

We hypothesise that apathy and anhedonia can be reliably identified and tracked using ECOCAPTURE digital biomarkers. Specifically, we propose that apathy markers will exhibit momentary fluctuations contingent on context and environment, whereas anhedonia will remain relatively stable over the course of the day.

**Conclusions:**

This project will contribute novel objective behavioural indices of BPSDs that can be effectively implemented in the home environment. These digital biomarkers will provide essential information to identify windows of opportunity for intervention. Moreover, our approach will enable therapeutic monitoring of targeted interventions supporting patients’ autonomy, alleviating caregivers’ stress, and enabling people with dementia to age in place.

